# Surgical treatment of gastric cancer: a 10-year experience in a high-volume university hospital

**DOI:** 10.6061/clinics/2018/e543s

**Published:** 2018-11-27

**Authors:** Marcus Fernando Kodama Pertille Ramos, Marina Alessandra Pereira, Osmar Kenji Yagi, Andre Roncon Dias, Amir Zeide Charruf, Rodrigo Jose de Oliveira, Evelise Pelegrinelli Zaidan, Bruno Zilberstein, Ulysses Ribeiro-Júnior, Ivan Cecconello

**Affiliations:** Instituto do Cancer do Estado de Sao Paulo (ICESP), Hospital das Clinicas HCFMUSP, Faculdade de Medicina, Universidade de Sao Paulo, Sao Paulo, SP, BR

**Keywords:** Gastric Cancer, Gastrectomy, Operative Surgical Procedures

## Abstract

**OBJECTIVES:**

Surgery remains the cornerstone treatment modality for gastric cancer, the fifth most common type of tumor in Brazil. The aim of this study was to analyze the surgical treatment outcomes of patients with gastric cancer who were referred to a high-volume university hospital.

**METHODS:**

We reviewed all consecutive patients who underwent any surgical procedure due to gastric cancer from a prospectively collected database. Clinicopathological characteristics, surgical and survival outcomes were evaluated, with emphasis on patients treated with curative intent.

**RESULTS:**

From 2008 to 2017, 934 patients with gastric tumors underwent surgical procedures in our center. Gastric adenocarcinoma accounted for the majority of cases. Of the 875 patients with gastric adenocarcinoma, resection with curative intent was performed in 63.5%, and palliative treatment was performed in 22.4%. The postoperative surgical mortality rate for resected cases was 5.3% and was related to D1 lymphadenectomy and the presence of comorbidities. Analysis of patients treated with curative intent showed that resection extent, pT category, pN category and final pTNM stage were related to disease-free survival (DFS) and overall survival (OS). The DFS rates for D1 and D2 lymphadenectomy were similar, but D2 lymphadenectomy significantly improved the OS rate. Additionally, clinical factors and the presence of comorbidities had influence on the OS.

**CONCLUSIONS:**

TNM stage and the type of lymphadenectomy were independent factors related to prognosis. Early diagnosis should be sought to offer the optimal surgical approach in patients with less-advanced disease.

## INTRODUCTION

Gastric cancer (GC) is the fifth most common cancer worldwide. Approximately one million (952,000) new cases of GC occurred worldwide in 2012 1. In Brazil, it is estimated 12,920 new cases of stomach cancer occurred in men and 7,600 occurred in women in the biennium 2016–2017 2. Adenocarcinoma is the most frequent histological type of GC, accounting for more than 95% of tumors. Other histological types are gastrointestinal stromal tumor (GIST), leiomyoma, lymphoma, and neuroendocrine tumor 3.

Surgery remains the main curative option of GC. Gastrectomy with D2 lymphadenectomy is considered the standard surgical treatment for locally advanced GC; this modality is characterized by tumor resection with adequate margins along with removal of the lymph nodes at the origin of the main gastric vessels. A more limited D1 lymphadenectomy may be indicated for frail patients with multiple comorbidities. Furthermore, endoscopic treatment may be an option for early tumors with a low risk of lymph node metastasis 4.

Unfortunately, not all patients are candidates for resection with curative intent. A substantial number of patients undergo noncurative procedures to palliate symptoms such as obstruction and/or bleeding. Moreover, diagnostic surgical procedures may be employed to assess peritoneal dissemination and define the purpose of chemotherapy as either neoadjuvant or palliative 4.

The aim of this study was to analyze the characteristics and outcomes of patients with gastric tumors who were referred to surgical treatment at a high-volume university hospital. Clinicopathological characteristics, surgical and survival outcome were evaluated with emphasis on patients treated with curative intent.

## METHODS

We reviewed all consecutive patients who underwent any surgical procedure due to GC from 2008 to 2017 at Cancer Institute, University of Sao Paulo Medical School. The data were prospectively collected and entered into a clinical database. Patients with tumors not originating in the stomach or who underwent gastric surgical procedures related to benign conditions such as peptic ulcer or gastrostomy were excluded from the analysis.

Patients were staged preoperatively through abdominal and pelvic computed tomography, upper digestive endoscopy and laboratory tests. The intent of surgery and surgical technique followed the recommendations of the Japanese Gastric Cancer Association guidelines 4. Patients who had previously undergone diagnostic or endoscopic procedures but subsequently underwent resection were included in the resection group. The extent of the gastric resection (total × subtotal) was based on the location of the tumor in order to obtain a tumor-free proximal margin 5. Resection was defined as R0, R1 or R2 according to the amount of residual tumor (no residual tumor, microscopic or macroscopic, respectively). The extent of nodal dissection (D1 or D2 lymphadenectomy) was defined according to the lymph node chains removed. TNM staging was performed according to the 7^th^ edition 6.

All surgeries were performed by surgeons with extensive experience in the surgical management of GC. Some emergency procedures were performed by other gastrointestinal surgeons at the hospital. Surgical residents played an active role during most procedures. Complex cases were discussed in the weekly multidisciplinary meeting.

Clinical characteristics, including *American Society of Anesthesiologists* (ASA) classification 7, *Charlson–Deyo comorbidity index* (CCI) 8, and laboratory test results, were evaluated. The CCI was evaluated without the inclusion of age and GC neoplasm as a comorbidity.

Surgical complications were graded according to the *Clavien–Dindo* classification 9. Major complications were considered *Clavien* III–V. The hospitalization period and the number of retrieved lymph nodes were evaluated. Surgical mortality was considered when death occurred within the first 30 days after surgery or during the hospital stay after the procedure.

Postoperative follow-up was performed on a quarterly basis in the first year after surgery and every six months in the following years. Follow-up studies for relapse detection were performed based on the presence of symptoms. Absence from medical appointments for more than 12 months was considered loss to follow-up.

The study is part of a larger project, “Banco de dados de pacientes do Serviço de Cirurgia do Aparelho Digestivo do ICESP-HCFMUSP”, which was approved by the hospital ethics committee (NP993/16) and registered in the “Plataforma Brasil” (CAAE: 2915516.2.0000.0065).

### Statistical analysis

The descriptive statistics included frequencies with percentages for nominal variables and means for continuous variables. To evaluate the differences between the variables, chi-square tests were used for categorical data; *t*-tests, for continuous data. Disease-free survival (DFS) and overall survival (OS) were estimated using the Kaplan–Meier method, and differences in survival were compared using the log-rank test. To determine factors associated with DFS and OS, we used univariate and multivariate Cox proportional hazards regression models. Variables significant in the univariate analysis were included as covariables in the multivariable analysis to determine which variables independently affected prognosis. Survival time was calculated from the date of surgery until the date of death/recurrence. Surviving patients were censored at the date of last contact. All tests were two-sided, and statistical significance was defined as *p<*0.05. Analyses were performed using SPSS software, version 18.0 (SPSS Inc, Chicago, IL).

## RESULTS

A total of 934 patients were surgically treated for primary gastric tumors between 2008 and 2017 in our institution. Gastric adenocarcinoma was diagnosed in 875 patients (93.7%). Nonadenocarcinoma primary gastric tumors, including GIST (n=31, 3.3%), neuroendocrine tumor (n=10, 1.1%) and lymphoma (n=8, 0.9%), were detected in 59 patients (6.3%). Ten patients (1.1%) presented benign tumors, such as leiomyoma, lipoma and schwannoma. Nonadenocarcinoma tumors were not included in further analyses.

Clinical and surgical characteristics of the remaining 875 gastric adenocarcinoma patients are shown in Table 1. The mean age at initial diagnosis was 63.5 years (range 23–95 years), and 81 patients (9.3%) were older than 80. The proportion of male patients was higher than female patients, with a male–female ratio of 1.7, and the mean body mass index (BMI) of the cohort was 23.7 kg/m^2^. The predominant CCI was 0-1, and the ASA preoperative risk classification was I/II for the majority of the patients (72.9%).

Of these patients, 63.5% were treated with curative intent, while 22.4% underwent palliative surgery. Regarding tumor location, 53.9% of the lesions were located in the distal stomach. Among the patients who underwent resection, the residual tumor category was R0 in 68.9%, R1 in 1.4%, and R2 in 29.7%. Regarding the UICC TNM stage classification, cTNM stage III was the most frequent (32.2% of patients).

### Survival analysis – Gastric adenocarcinoma patients

**Figure 1:**
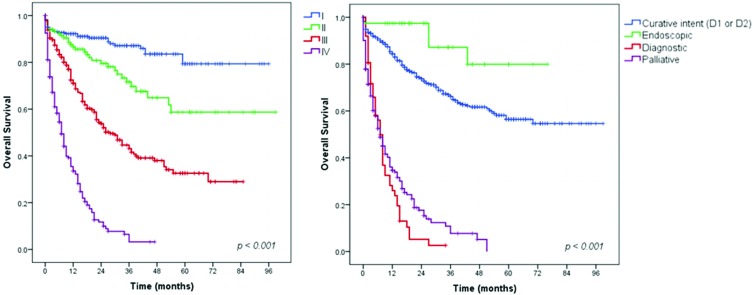
Survival curves for gastric adenocarcinoma patients according to cTNM stage and surgical intent.

All adenocarcinoma patients were included in the first survival analysis. The mean follow-up duration was 28 months (range 0-99 months). During follow-up, 45.2% of the patients had disease recurrence, and 360 died. A total of 110 (12.6%) patients were lost during follow-up. The DFS and OS rates for the entire cohort were 54.8% and 58.9%, respectively. Figure 1 shows the OS rate for the patients according to cTNM stage and treatment intent. For the different stages, the OS rate was 87.3% for stage I, 76.5% for stage II, 52.8% for stage III, and 27.2% for stage IV disease. Patients who underwent endoscopic treatment, followed by patients who underwent resection with curative intent, had higher survival rates than patients who underwent other treatments (90.5% and 71.2%, respectively). Patients who received palliative (79.7% were staged as IV) or diagnostic procedures (77.9% were staged as IV) had survival rates of 28.2% (median OS= 8 months, 95% CI 6.16–9.84 months) and 27.9% (median OS= 7 months, 95% CI 5.60–8.40 months), respectively, with no patients alive at the 5-year follow-up.

### Clinicopathological features – Gastrectomy with curative intent group

To better assess the pathological characteristics and survival outcomes of patients with GC treated at our institution, we further analyzed only the 509 patients with gastric adenocarcinoma who underwent surgical resection with curative intent. Patients with gastric remnant tumors were not included. The clinicopathological characteristics of these patients are shown in Table 2.

The mean age was 62.9 years, and male patients accounted for 304 cases (59.7%). The mean BMI at the time of surgery was 24.6 kg/m2 (range 13–57), and the mean albumin and hemoglobin levels were 4.0 mg/dL and 12.5 g/dL, respectively. Neoadjuvant therapy was performed in 64 (12.6%) patients.

The main tumor location was the lower third of the stomach, and subtotal gastrectomy with D2 lymphadenectomy was the most common procedure performed. Forty-five (8.8%) surgeries were performed laparoscopically, and 22 (4.3%) were robotic surgeries. Total gastrectomy was performed more frequently for pT3/T4 tumors than for pT1/T2 tumors (67.7% *vs.* 33%, *p*=0.008).

Regarding the pT category, subserosal layer invasion occurred in 31.6% of the patients. Lymph node metastases were present in 55.2% of the cases, with a mean of 4.9 (range 0–67) positive lymph nodes per patient. The mean number of lymph nodes retrieved in the surgical specimens over all patients was 39.5. Regarding the type of lymphadenectomy, a mean of 41.8 lymph nodes were obtained in D2 dissection and 27.7 in D1 dissection (*p<*0.001). Regarding the TNM classification, pTNM stage III was the most frequent stage (42% of cases).

The median length of hospital stay was 9 days (4–73 days). Minor complications (grades I and II) occurred in 94 patients (18.5%), while major complications happened in 44 (8.6%) patients. The postoperative mortality rate was 5.3% (27 patients). Mortality was higher for those who underwent D1 lymphadenectomy (n=13, 15.3%) than for those receiving D2 lymphadenectomy (n=13, 3.3%) (*p<*0.001). The main causes of death were pneumonia (7 of 20 patients), followed by duodenal stump fistula (5 of 15 patients), with surgical mortality rates of 35% and 33.3%, respectively. Complications were more frequent in elderly patients (>70 years= 20% *vs.* <70 years=11%, *p*<0.001), patients with higher CCI (0–1= 13.5% *vs.* >1= 17.3%, *p*=0.017) and patients with an higher ASA classification (I/II= 11.2% *vs.* III/IV= 24.1%, *p*<0.001).

Postoperative adjuvant chemotherapy and chemoradiotherapy were administered to 117 (23%) and 118 (23.2%) patients, respectively. During the follow-up period, the DFS and OS rates for the cohort treated with curative intent resection was 76.8% and 72.3%, respectively. Of the 110 patients with recurrence, the most frequent sites of recurrence were local/regional lymph nodes (67 patients, 60.9%), the peritoneum (43 patients, 39.1%) and distant sites (45 patients, 40.9%), with some patients presenting more than one site of recurrence. The main sites of distant metastasis were the liver (26 patients), lung (10 patients), bones (5 patients) and ovary (4 patients).

### Survival analysis – Gastrectomy with curative intent group

**Figure 2:**
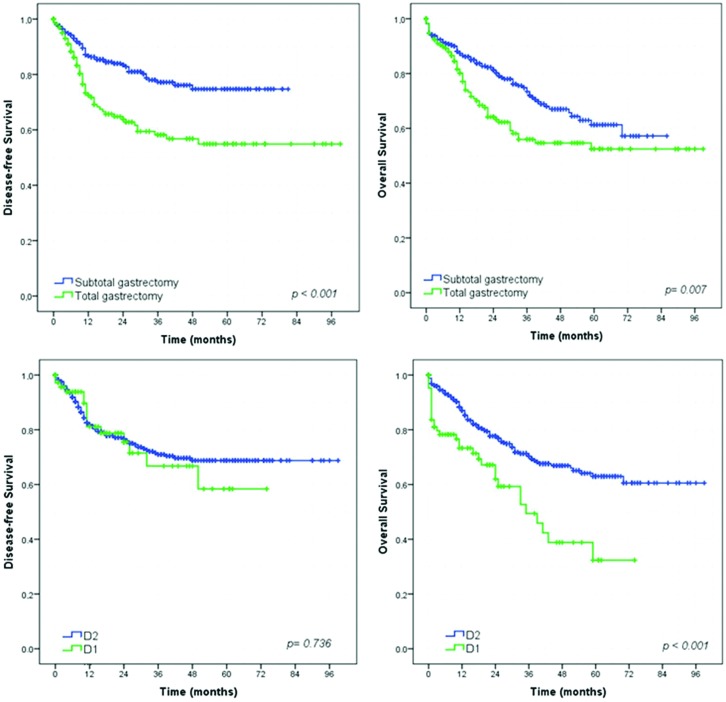
Survival curves according to the type of surgery and lymphadenectomy for patients who underwent curative resection.

**Figure 3:**
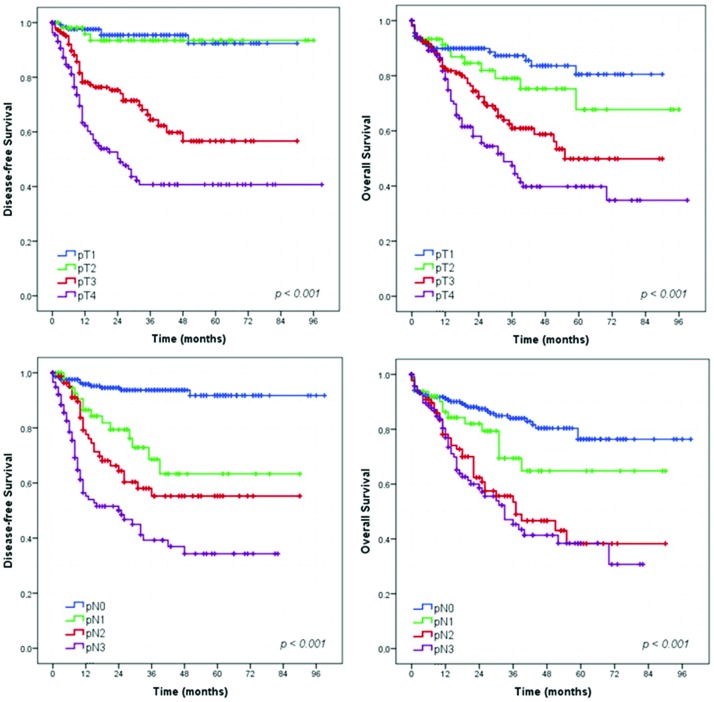
Survival curves according to depth of invasion and lymph node status for patients who underwent curative resection.

**Figure 4:**
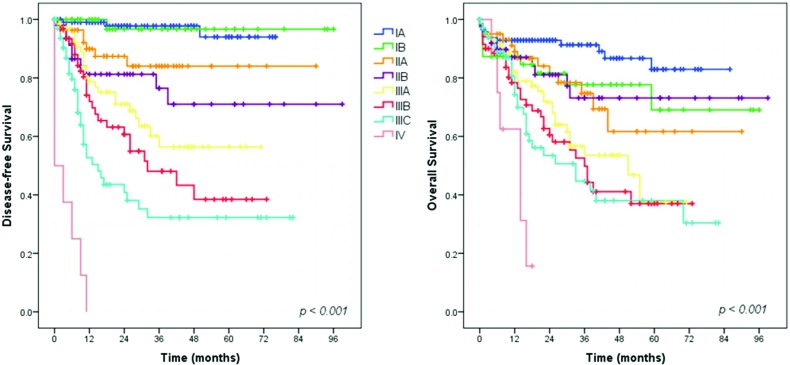
Survival curves according to pTNM stage for patients who underwent curative resection.

Survival analysis showed that extent of surgery (total gastrectomy), greater depth of tumor invasion (pT status), higher frequency of metastatic lymph nodes (pN status) and more advanced pTNM stage had a significant negative impact on DFS and OS (Figures 2, 3 and 4). The DFS rates for D1 and D2 lymphadenectomy were similar (*p=*0.736), but D2 lymphadenectomy significantly improved the OS rate (*p<*0.001).

In the DFS analysis, the log-rank test for pT category revealed a statistically significant difference between stages T1 and T3 (*p<*0.001), T1 and T4 (*p<*0.001), T2 and T3 (*p<*0.001) and T3 and T4 (*p=*0.001). However, no significant difference was found between stages T1 and T2 (*p=*0.801). In addition, there were statistically significant differences across the pN categories: between stages N0 and N1/N2/N3 (*p<*0.001 for all), N1 and N3 (*p<*0.001), and N2 and N3 (*p=*0.04). However, no significant difference was noted between stages N1 and N2 (*p=*0.176).

Regarding OS, the log-rank test for pT category revealed statistically significant differences between stages T1 and T3 (*p<*0.001), T1 and T4 (*p<*0.001), T2 and T4 (*p=*0.001) and T3 and T4 (*p=*0.024). However, no significant difference was found between stages T1 and T2 (*p=*0.244) or T2 and T3 (*p=*0.062). Across pN category, statistically significant differences were observed between stages N0 and N2/N3 (*p<*0.001 for both), N1 and N2 (*p=*0.030), and N1 and N3 (*p=*0.007). However, no significant difference was noted between stages N0 and N1 (*p=*0.085) or N2 and N3 stage (*p=*0.563). Although no significant difference was noted between some pT and pN categories, the cumulative survival curves for patients with different stages did not intersect (Figure 3).

The results of the multivariate analysis are shown in Table 3. Worse DFS was associated with type of gastrectomy, depth of invasion (pT) and lymph node metastasis (pN). Regarding OS, the multivariate analysis identified advanced age, ASA classifications III and IV, CCI >1, D1 lymphadenectomy, diffuse/mixed Lauren type, pT3/T4 and pN1/N2/N3 stage as independent predictors of poor prognosis.

## DISCUSSION

In the present study, we reported a broad view of GC surgical treatment based on a 10-year experience in a high-volume center. Gastric adenocarcinoma accounted for the vast majority of the cases. Resection with curative intent was performed in 63.5% of the patients. Resection extent, pT category, pN category and final pTNM stage were related to DFS and OS. OS was also influenced by the clinical factors and comorbidities of the patients.

Initial analysis of clinical staging can provide insight into the state in which patients eventually present at a reference center for treatment. A quarter of the cases were already stage IV, and the survival rates of patients not treated with curative intent are clearly lower than those treated with curative intent. Regarding the type of surgical procedure, endoscopic resection accounted for only a small percentage, and a substantial number of palliative procedures were performed. These findings highlight the need for improving public health policy to achieve earlier diagnosis of GC. Countries with a more active policy of screening and testing symptomatic patients have an incidence rate of early tumors of higher than 50% 10. Even though we expect to improve the rate of early diagnosis in the future, we were encouraged and surprised by the frequency of 34% for stage I classification in resected patients 11. Recent series from referral centers in Chile, China and the United States reported stage I frequencies of 19.3%, 28% and 50%, respectively 12,13.

Surgical mortality rate of resected patients was 5.3%, reflecting the high complexity of these procedures. The main causes of death were pneumonia and duodenal stump fistula, which was previously described as the most lethal surgical complication following gastrectomy 14. Complications and mortality were related to older patients with comorbidities who underwent D1 lymphadenectomy. Importantly, our criterion for performing D1 dissection is precisely restricted to patients with a high surgical risk. This association demonstrates the appropriate indication for a more limited procedure in high-risk patients. When we consider only D2 dissection, mortality drops to 3.3%, similar to that observed in other Western series 12,15-17. In addition to the type of lymphadenectomy, in the present study total gastrectomy was associated with a poorer prognosis compared to subtotal resection. This association may be related to the higher risk for serious postoperative complications after total gastrectomy and the different molecular subtypes of GC 18,19.

The mean number of harvested lymph nodes is used globally to compare and evaluate surgical quality. The mean number of lymph nodes obtained in D2 lymphadenectomy was 41.8, which greatly exceeds the minimum number of 16 recommended for an adequate TNM staging 6 and the minimum number of 25 recommended by the Brazilian Consensus Guidelines 5 to consider a D2 lymphadenectomy adequate. Even D1 cases with a mean of 27.7 lymph nodes harvested meet the requirements for an adequate lymph node dissection.

The oncological benefits of D2 dissection have been widely described in the literature 20,21. Even though, in our series, the DFS rates for D1 and D2 lymphadenectomy were similar (*p=*0.736), D2 lymphadenectomy significantly improved the OS. However, patients with poorer clinical conditions exhibit a limited expected survival time; therefore, they may not have time to benefit from D2 lymph node dissection. The balance between the patients comorbidities and the choice of lymphadenectomy in each individual case is a challenge for surgeons.

Stage II/III/IV patients, the target population for multimodal treatment according to the hospital protocol, accounted for 66% of the patients. However, only 46% of cases received any type of adjuvant therapy. Referral of patients to the medical oncology department at the hospital is fast and straightforward. The lack of provision of adjuvant treatment was probably due to the poor clinical performance of patients. Furthermore, the occurrence of tumors with low lymph node involvement in patients with well-performed surgical resection may have discouraged adjuvant therapy.

As expected, the greater the tumor invasion, the poorer was the prognosis. The DFS and OS significantly decreased from stage pT3 upward. Patients with tumors staged pT3 or pT4 had significantly poorer prognosis than those with tumors staged pT2. Similarly, the survival rates were markedly decreased for patients with increased numbers of metastatic lymph nodes. Patients with one or more positive lymph nodes had a statistically significantly poorer DFS than patients with negative nodes. Furthermore, OS was significantly poorer in patients with more than three metastatic lymph nodes (stage pN2 or higher).

Our survival analysis demonstrated a very sharp separation of the DFS curves when stratified by the pTNM stage. However, the same linearity was not seen for the OS curves. This finding can be explained by several reasons. First, many patients did not complete the recommended 5-year follow-up and, as recurrence happens first, the DFS curves become separated earlier. Second, some patients with GC are very frail and can die of other causes. Therefore, deaths during follow-up may occur despite TNM staging, thus influencing only the OS. Furthermore, loss to follow-up occurs more often after than before the diagnosis of recurrence, because some patients facing the end phase of the disease tend to return to their hometowns. Thus, we have information about recurrence events, but the date of death may be unknown.

Regardless of the underlying factors that influence survival, our analyses showed that curative gastric resection with extended lymphadenectomy, along with early detection of the cancer, markedly contributed to improving survival. Importantly, despite complete surgical clearance of the primary tumor (R0 resection), some patients still suffer local or distant disease recurrence (60.9% and 40.9%, respectively, in our cohort), possibly due to disseminated micrometastases present at the time of surgery 22.

The current study has some limitations. As the objective of the study was to give a broad view of GC treatment with the description of the entire population, some variables and subgroups were not deeply analyzed. We did not consider the type of adjuvant therapy, which may influence survival by the administration of different regimens with different schedules. In addition, multivariate analyses were performed only with patients treated with curative intent, and stage subgroups such as T1a/T1b, T4a/T4b and N3 categories were presented as a single category (pT1, pT4 and pN3), and were not considered separately in the survival analyses. The surgical morbimortality analysis was limited to patients treated with curative resection. Last, some included patients have not yet reached the follow-up time of 5 years.

To our knowledge, this series is one of the largest to report the analysis of the clinicopathological characteristics and survival of patients with GC in Brazil. All patients were treated in a referral oncology hospital by surgeons dedicated to GC surgery. This inclusion criterion improves the accuracy and homogeneity of the perioperative clinical data. In addition, survival outcome is based on a cohort of patients with a high number of examined lymph nodes, which minimizes the risk for understaging. Furthermore, follow-up was available for 765 (87.4%) of the 875 patients. Through the inclusion of all patients who underwent any surgical procedure, a broad view of current GC surgical management can be obtained while minimizing selection bias. 

In conclusion, these results demonstrate the relevance of awareness of surgical outcomes, survival and factors associated with prognosis in clinical settings. These data convey prognostic information that assists in the selection of different surgical modalities and clarify the surgical risks involved in the treatment to determine the best therapeutic strategy for GC patients. Early diagnosis should be sought since it is evident the relationship between disease stage and prognosis, in addition to the low survival rates of patients who cannot be treated with surgery.

## AUTHOR CONTRIBUTIONS

Ramos MF was responsible for the study design, data retrieval, critical analysis and manuscript drafting. Pereira MA was responsible for the data retrieval, statistical analysis, and manuscript drafting. Yagi OK, Dias AR, Charruf AZ, Oliveira RJ and Zaidan EP were responsible for the data retrieval and manuscript review. Zilberstein B, Ribeiro-Junior U and Cecconello I were responsible for the critical analysis and manuscript review.

## Figures and Tables

**Table 1:** Clinical and surgical characteristics of patients with gastric adenocarcinoma. All patients (n=875).

Variables	n	%
Age (years)		
Mean (range)	63.5 (23-95)	
Sex		
Male	550	62.9
Female	325	37.1
Charlson-Deyo Comorbidity Index (CCI)*		
0–1	770	88
>1	83	9.5
American Society of Anesthesiologists (ASA) Classification		
I/II	638	72.9
III/IV	237	27.1
Type of Surgery		
Curative intent (D1 or D2)	556	63.5
Palliative	196	22.4
Diagnostic	68	7.8
Endoscopic	42	4.8
Cytoreduction/salvage surgery	13	1.5
Tumor Location		
Upper/middle	195	22.3
Lower	472	53.9
Entire	15	1.7
Anastomosis	41	4.7
Not available	152	17.4
TNM Stage		
I	229	26.2
II	136	15.5
III	282	32.2
IV	228	26.1

*Some cases were not assessed for this status due to the absence of the relevant information in the original medical report.

**Table 2:** Clinicopathological characteristics of gastric adenocarcinoma patients after curative intent resection (n=509).

Variables	n	%
Age (years)		
Mean (range)	62.9 (23 - 95)	
Sex		
Male	304	59.7
Female	205	40.3
Charlson-Deyo Comorbidity Index (CCI)*		
0–1	444	87.2
>1	52	10.2
American Society of Anesthesiologists (ASA) Classification		
I/II	401	78.8
III/IV	108	21.2
Type of Resection		
Subtotal gastrectomy	330	64.8
Total gastrectomy	179	35.2
Lymphadenectomy		
D2	425	83.5
D1	84	16.5
Tumor Location		
Upper	38	7.5
Middle	105	20.6
Lower	318	62.5
Entire	10	2.0
not available	38	7.5
Tumor Size (cm)		
Mean	4.85	
Lauren Type*		
Intestinal	263	51.7
Diffuse/mixed	223	43.8
Undetermined	20	3.9
pT Status		
pT1/is	146	28.7
pT2	62	12.2
pT3	161	31.6
pT4	140	27.5
Harvested Lymph Nodes		
Mean (SD)	39.5	
pN Status		
pN0	228	44.8
pN1	68	13.4
pN2	89	17.5
pN3	124	24.4
TNM Stage		
I	173	34,0
II	114	22.4
III	214	42.0
IV	8	1.6

*Some cases were not assessed for this status due to the absence of the relevant information in the original medical report.

**Table 3:** Univariate and multivariate analysis of disease-free survival and overall survival.

Disease-free Survival	Univariate			Multivariate		
Variables*	Hazard Ratio	95% CI	*p*	Hazard Ratio	95% CI	*p*
Age 0-69 *vs.* ≥70 years	0.69	0.44-1.07	0.095	—	—	—
Female *vs.* Male	1.07	0.73-1.58	0.710	—	—	—
ASA I/II *vs.* III/IV	0.91	0.54-1.53	0.733	—	—	—
Charlson 0-1 *vs.* Charlson >1	0.87	0.44-1.72	0.693	—	—	—
Subtotal *vs.* Total gastrectomy	2.16	1.48-3.14	<0.001	1.83	1.26-2.67	0.002
D2 *vs.* D1 lymphadenectomy	1.10	0.64-1.89	0.737	—	—	—
Intestinal *vs*. Diffuse/mixed	1.91	1.30-2.79	0.001	1.44	0.98-2.17	0.062
pT1/pT2 *vs.* pT3/pT4	10.03	5.07-19.85	<0.001	4.6	2.20-9.68	<0.001
pN0 *vs.* pN1/2/3	8.48	4.65-15.46	<0.001	3.68	1.99-7.08	<0.001

Overall Survival	Univariate			Multivariate		
Variables*	Hazard Ratio	95% CI	*p*	Hazard Ratio	95% CI	*p*
Age 0-69 *vs* ≥70 years	1.50	1.07-2.11	0.019	1.44	1.01-2.06	0.044
Female *vs*. Male	1.18	0.84-1.66	0.352	—	—	—
ASA I/II *vs.* III/IV	2.08	1.44-3.01	<0.001	1.68	1.10-2.56	0.016
Charlson 0-1 *vs*. Charlson >1	1.54	0.94-2.53	0.089	—	—	—
Subtotal *vs*. Total gastrectomy	1.58	1.13-2.21	0.008	1.61	1.14-2.25	0.006
D2 *vs.* D1 lymphadenectomy	2.19	1.48-3.24	<0.001	1.99	1.26-3.13	0.003
Intestinal *vs.* Diffuse/mixed	1.49	1.06-2.07	0.002	1.45	1.03-2.05	0.034
pT1/pT2 *vs.* pT3/pT4	2.92	1.96-4.36	<0.001	1.91	1.18-3.11	0.009
pN0 *vs.* pN1/2/3	3.03	2.06-4.47	<0.001	2.04	1.27-3.29	0.002

*The first variable represents the reference category.
